# Chorioamnionitis, Inflammation and Neonatal Apnea: Effects on Preterm Neonatal Brainstem and on Peripheral Airways: Chorioamnionitis and Neonatal Respiratory Functions

**DOI:** 10.3390/children8100917

**Published:** 2021-10-15

**Authors:** Giovanna Vitaliti, Raffaele Falsaperla

**Affiliations:** 1 Unit of Pediatrics, Department of Medical Sciences, Section of Pediatrics, University of Ferrara, 44121 Ferrara, Italy; 2 Pediatrics and Pediatric Emergency Operative Unit, Azienda Ospedaliero Universitaria Policlinico G.Rodolico-San Marco, San Marco Hospital, University of Catania, 95124 Catania, Italy; raffaelefalsaperla@hotmail.com; 3 Neonatal Intensive Care Unit, Azienda Ospedaliero Universitaria Policlinico G.Rodolico-San Marco, San Marco Hospital, San Marco Hospital, University of Catania, 95124 Catania, Italy

**Keywords:** inflammation, chorioamnionitis, brainstem, cytokines storm, preterm birth, neonatal apnea, apnea physiopathology

## Abstract

**Background:** The present manuscript aims to be a narrative review evaluating the association between inflammation in chorioamnionitis and damage on respiratory centers, peripheral airways, and lungs, explaining the pathways responsible for apnea in preterm babies born by delivery after chorioamnionitis. **Methods:** A combination of keywords and MESH words was used, including: “inflammation”, “chorioamnionitis”, “brainstem”, “cytokines storm”, “preterm birth”, “neonatal apnea”, and “apnea physiopathology”. All identified papers were screened for title and abstracts by the two authors to verify whether they met the proper criteria to write the topic. **Results:** Chorioamnionitis is usually associated with Fetal Inflammatory Response Syndrome (FIRS), resulting in injury of brain and lungs. Literature data have shown that infections causing chorioamnionitis are mostly associated with inflammation and consequent hypoxia-mediated brain injury. Moreover, inflammation and infection induce apneic episodes in neonates, as well as in animal samples. Chorioamnionitis-induced inflammation favors the systemic secretion of pro-inflammatory cytokines that are involved in abnormal development of the respiratory centers in the brainstem and in alterations of peripheral airways and lungs. **Conclusions:** Preterm birth shows a suboptimal development of the brainstem and abnormalities and altered development of peripheral airways and lungs. These alterations are responsible for reduced respiratory control and apnea. To date, mostly animal studies have been published. Therefore, more clinical studies on the role of chorioamninitis-induced inflammation on prematurity and neonatal apnea are necessary.

## 1. Introduction

Preterm birth is considered when childbirth occurs <37 weeks of gestation and represents an important risk factor for neonatal morbidity and mortality worldwide [1], with an average of 15 million preterm births annually and 1.1 millions infants who die from various complications [2].

Causes of preterm birth are multifactorial, including fetal/maternal abnormalities [3] and environmental factors [3].

Interestingly, more than 60% of preterm infants with a gestational age (GA) <28 weeks are exposed to chorioamnionitis [4], with the disease the being considered the most important antecedent of prematurity [5,6,7].

In the transition from intra- to extrauterine life, processes of complex adaptation to environmental factors that assure an effective shift from maternal dependence to neonatal autonomy occur [5,6,7].

A primary physiological event to succeed in this extrauterine transition is lung aeration [8], and this is possible by a proper clearance of lung liquid, normal surfactant production, and variations in cardiovascular resistances with increasing blood flow, which occur upon birth [8]. At this time, the autonomous nervous system and the brainstem should be fully functional, with its vital respiratory centers responsible for rhythm generation and breathing biomechanics coordination [9].

Preterm neonates frequently develop a respiratory distress syndrome (RDS) upon delivery [10], which is attributed to insufficient lung liquid clearance, immature development of their lungs, and surfactant deficiency, with subsequent failure in respiration, inadequate gas exchanges, and apneic episodes [11].

When a preterm infant presents with RDS, an emergent respiratory support at delivery is mandatory, with subsequent ventilation during the transfer to the neonatal intensive care unit (NICU) [12].

Further, preterm babies who are exposed to inflammation and/or infection causing chorioamnionitis usually need greater requirement for respiratory support, with a greater risk to develop severe neurological damage with respect to those neonates who are not exposed to chorioamnionitis [13,14,15,16,17].

Chorioamnionitis is defined as a perinatal condition presenting with inflammation of the fetal membrane, including the chorion and the amnion [13,14,15,16,17]. The clinical presentation of this disease can vary based on microbiologic, histologic, and clinical features, which interact among one another to varying degrees [13,14,15,16,17].

Acute chorioamnionitis is an expression of maternal host response. Intraamniotic infection generally has been found to be the main cause of acute chorioamnionitis; however, recent studies indicate that “sterile” intraamniotic inflammation, without demonstrable microorganisms inducing infection, is frequently associated with acute chorioamnionit. In the context of intraamniotic infection, pro-inflammatory cytokines and chemokines establish a gradient that allows the migration of neutrophils and other immune cells from the maternal or fetal circulation into the chorioamniotic membranes [17].

Literature data have widely demonstrated the link between inflammation and periventricular leukomalacia (PVL), cerebral hemorrhage, and post-hemorrhagic hydrocephalus (PHH); nevertheless, the effects of inflammation on the development of the autonomous nervous system and the brainstem centers responsible for breathing regulation remain largely unknown [5].

This review of literature data aims to study the effects of inflammation caused by chorioamnionitis on respiratory centers and on peripheral airways and lungs, explaining the pathways responsible for apnea in preterm babies born by delivery after chorioamnionitis.

## 2. Materials and Methods

Articles reporting evidence on the association among “inflammation”, “chorioamnionitis”, and “neonatal apnea” were selected. Multiple databases were combined for a comprehensive literature search. The literature search was performed in MEDLINE via PubMed interface, SCOPUS, the Cochrane Library, and Google Scholar, for all papers published from inception to September 2021. Database-specific search strings were developed including search terms describing inflammation in chorioamnionitis as a cause of preterm birth (population/exposing factors), and signs or symptoms of apnea, especially in preterm infants (study design/single case reports/case series), as dysregulation of the autonomous nervous system. A combination of MESH words and keywords was used, including the following ones: “inflammation”, “chorioamnionitis”, “dysautonomia signs”, “brainstem”, “cytokines storm”, “preterm birth”, “neonatal apnea”, and “apnea physiopathology”.

All identified papers were screened for titles and abstracts by the two authors: The two authors studied all included papers to determine whether they met the inclusion criteria to develop the selected topic. Moreover, full texts of included articles were independently retrieved by the two authors for eligibility.

## 3. Results

Three-hundred-and-eighty titles and abstracts were screened; 210 were excluded because they did not center the topic; 170 full text were read; and 90 were excluded for incomplete details. In this review, 80 articles were included to develop the discussion.

## 4. Discussion

Inflammation and infection of the chorionic membrane (including the amniotic fluid) is defined as chorioamnionitis [5]. This condition is frequently caused by infection of a maternal ascending polymicrobial pathway and can involve the fetus when exposed to the infection by direct contact with amniotic fluid, or via placental-fetal circulation [17].

Different bacterial, viral, and fungal organisms have been recognized as responsible for chorioamnionitis, and ureaplasmas is often isolated as a responsible organism [18]. The diagnosis of chorioamnionitis can be performed after birth by analysis of the placenta, and to date there is insufficient therapeutic intervention to reduce inflammation during gestation; thus, in this condition, the fetal vital organs can be seriously damaged [5].

Chorioamnionitis is usually associated with Fetal Inflammatory Response Syndrome (FIRS), which is defined by increased systemic inflammatory cytokines (for e.g., interleukin-6) concentrations, funisitis, and fetal vasculitis, with possible secondary extensive damage of lungs and brain [16,17,19,20]. Literature data have shown that infections causing chorioamnionitis are often associated with inflammatory and hypoxia-mediated brain damage in both neonates and animal models [20,21,22,23,24,25]. Among neurologic diseases following chorioamnionitis, neurodevelopmental diseases, poor cognitive outcome, as well as poor behavioral and neuromotor development in infants, have been described and are implicated in 11–22% of cerebral palsy [15,26,27,28].

Moreover, inflammation and infection induce apneic episodes in neonates, as well as in animal models [29,30,31,32,33]. Thus, chorioamnionitis is often associated with recurrent apnea in preterm infants, SIDS known as “sudden infant death syndrome,” and obstructive sleep apnea syndrome in ex-preterm children [34,35,36,37,38].

Antenatal corticosteroids are used as a therapeutic strategy to improve lung maturation in preterm neonates [39,40]. Steroids seem to be effective in reducing severity chorioamnionitis by histological findings and in reducing the severity of RDS [39,40]. Nevertheless, studies on the effects of steroids (at single or repeated doses) on auditory brainstem pathways in preterm infants and animals with and without chorioamnionitis have given different results [41,42,43]. In fact, in mice models, repeated doses of steroids negatively affected neural transmission and auditory brainstem responses [43]. Furthermore, repeated dosed of steroids in fetal sheep caused a decrease in cerebral weight without involving the cerebellum pathways [44]. In this regard, we have to mention that on one hand steroids have an anti-inflammatory effect, on the other hand they display an important key-role in immunosuppression. Therefore, in chorioamnionitis it has to be established whether the origin is predominantly of inflammatory or infective nature, as in the second case steroids should not be advisable.

Moreover, the actions of steroids on the brainstem respiratory centers are still unknown. The exact mechanisms causing changes in respiratory function are not clearly understood, but new literature data highlights a key-role for inflammatory cytokines and prostaglandins in producing alterations of the respiratory function [5].

### 4.1. The Link between Chorioamnionitis and Brainstem Function

Literature data showed that lipopolysaccharide-LPS (cell wall constituent of Gram-negative bacteria) is used to model chorioamnionitis in animal studies [16,45,46]. This bacterial endotoxin is responsible for important and reproducible inflammatory responses. It is frequently used to reproduce chorioamnionitis in vitro.

LPS is a ligand for Toll-like receptors (TLR), which stimulate downstream signaling pathways, thus inducing inflammatory cytokines production [47,48]. LPS especially binds to TLR4, activating a pathway that results in interferon (IFN)-related cytokines production, and potentiating the gene transcription of the nuclear factor kappa-light-chain-enhancer of activated B cells (NF-kB) [49]. This transcription is responsible for the secretion of interleukins including: IL-1b, IL-6, and IL-8; Tumor Necrosis Factors: TNF-a, TNF-b; inducible cyclooxygenase (COX-2 favor prostaglandin synthesis); and inducible nitric oxide synthase (iNOS) [50,51,52].

TLRs are also expressed in microglia and astrocytes within the brain, playing an important role in cytokine production [53].

### 4.2. Proinflammatory Cytokines and the Brainstem

Literature data have recently demonstrated that inflammation and consequent “cytokine storm” are responsible for alterations of the brainstem, with consequent abnormal function of the respiratory center, which finally results in episodes of apnea. This system is reproducible also for neonatal apnea of the premature when exposed to chorioamnionitis.

In rats brainstem, LPS exposure upregulates IL-1b and IL-6 mRNA expression [54] with consequent alteration of neuronal function within the pre-Botzinger Complex (pBTOC), whose neurons are included in the respiratory group of the ventral area of the medulla oblongata [54].

Electrophysiological traces of pre-Botzinger neurons in the pBOTC from neonatal mice after intrauterine LPS injection caused changes in the functions of pacemaker neurons, showing large amplitude bursts, at irregular and slow frequency [55].

Studies showed that IL-1b and IL-6 depress inhibitory synaptic signaling and contemporarily increase excitatory signaling within the pBOTC, thus explaining the onset of longer inspiratory drive and absent respiratory activity. These processes are then responsible for apnea in the neonate [55].

Neuronal activity can be rapidly modified by cytokines, with changes that persist long-term [56]. IL-1b, IL-6, and TNF-a can modulate neuronal functions of the central nervous system (CNS) by enhancing signaling of excitatory pathways and depressing inhibitory ones [57]. These pro-inflammatory cytokines alter neuronal excitability causing post-translational modification of the receptor of GABAergic, glutaminergic, and glycinergic nature, and affecting synaptic plasticity and neurotransmission [56,57].

In vitro and animal studies showed that IL-1b alters neuronal function according to its blood concentrations; at low concentration, IL-1 inhibits voltage-gated calcium currents and decreases calcium intracellular concentrations by inhibition of neurotransmitters release. At high concentrations, IL-1b increases inotropic glutamate (NMDA) receptor expression, and in rat cerebellar and hippocampal slices, reduces transmission at GABAergic and glycinergic receptors [56,57,58,59].

TNF-a alters neuronal excitability through the upregulation of a-amino-3-hydroxy-5-methyl-4-isoxazolepropionic acid (AMPA) and NMDA receptors; in rat hippocampus and cerebellum cultures, TNF-a induces gamma-aminobutirric acid (GABA) receptor endocytosis [57,60,61]. This causes an increase in excitatory output, with consequent excitotoxicity, as well as a decrease in inhibitory pathways. IL-6 seems to also have both destructive and protective functions within the CNS. Literature data showed that IL-6 induces a decrease in the expression of metabotropic glutamate receptor, and thus also induces excitotoxicity by excessively activating NMDA receptors [59,62,63,64]. Moreover, IL-6 has also shown the ability to reduce GABAergic and glycinergic neurotransmission in neurons of the dorsal horn of spinal cord in rat models [65].

It is now established that systemic inflammation/infection induced by LPS injection can alter chemosensory responses and breathing frequency, however, the pathways responsible for these modifications in respiratory functions are still not completely understood [66].

An imbalance between excitatory and inhibitory neurotransmission signaling has been previously shown in the brainstem of rats and piglets under hypoxic conditions [44,67,68,69], but little is known about the effects of inflammation and inflammatory molecules on neurotransmission within the brainstem. As brainstem neurons of the respiratory nuclei utilize GABA, glutamate, and glycine, cytokines involved in inflammation could affect the balance between excitatory and inhibitory neurotransmitters, and thus, cause consequent changes of neuronal function and respiratory responses [5].

Systemic injection of IL-1b, IL-6, and TNF-a induces the expression of their own mRNA within rat nucleus tractus solitarius (NTS) [70]. IL-1b injection into the rat NTS increases inspiratory time about 20 min after, and delays respiratory activity (usually after 80 min) [71]. Furthermore, IL-1b injection causes a reduction in respiratory frequency and induces apneustic episodes [71].

To date, it is unclear whether IL-1b causes modifications in neurotransmission within the NTS respiratory centers with consequent modulation of the respiratory rhythm, or whether it does so involving other downstream pathways. Robust IL-1b-immunoreactivity has been described in animal NTS and area postrema after LPS injection [54]. Previous studies showed that vagotomy can abrogate the NTS increase of IL-1b mRNA expression [54].

Administration of IL-1b to animal lungs and peritonea attenuates hypoxic and hypercapnic responses [54,72], suggesting that chemosensory reflexes are impaired in response to inflammatory stimuli. Literature data have still to clear whether altered chemosensory responses are secondary only to compromised central chemoreceptor functions, or whether modifications to vagal afferent signaling to the NTS are also responsible for these abnormalities, with consequent impaired diffusion of peripheral chemosensory information to brainstem respiratory centers by NTS neurons of second-order. It seems that inflammation/infection would induce robust changes to both peripheral and central chemoreflexes.

In addition to IL-1b expression in the NTS, literature data showed that LPS administration can cause robust immunoreactivity in the area postrema [54]. Studies have also described that the loss of blood brain barrier integrity plays a key-role in brain inflammation and injury as a consequence of systemic inflammatory cytokines and endotoxin exposure [73]. However, the NTS has connections with the area postrema, this last area representing a circumventricular region of the brainstem that could function as an entrance for inflammation.

In rat models, LPS injection also induces c-Fos immunoreactivity in neurons of the rostral ventrolateral medulla, NTS, and the respiratory nuclei [74], suggesting that neurons from these respiratory nuclei are reactive to inflammation/infection. Nevertheless, the exact mechanism for how (and which) inflammatory mediators modify the functions of neurons related to breathing has not been described [5].

Table 1 shows a resume of the activity of proinflammatory cytokines on the brainstem and how they modulate the respiratory centers to cause apnea. As shown in Table 1, studies have been performed in animal models, and clinical literature data are missing. Therefore, further studies on human models have to confirm these results.

### 4.3. Prostaglandin Effects on the Brainstem

It is well-established that mitogen-activated protein kinases (MAPK) and NF-kB signaling can induce COX isozyme expression. This event causes elevated PGs synthesis [75].

COX-1 and COX-2 are the two main COX isoforms. The first is constitutively expressed; the second is induced by tissue inflammation and injury. COX isozymes convert arachidonic acid prostaglandin H2 (PGH2) (precursor of the arachidonic acid) [76]. PGH2 is involved in the synthesis of PGE2, PGD2, PGF2a, and prostacyclin [75]. In detail, microsomal prostaglandin E2 synthase-1 (mPGES-1) catalyzes the synthesis of PGE2 from PGH2 [77]. Literature data have shown that COX2-mediated PGE2 production is involved in the inflammation of neonatal brain, especially in preterm babies, causing abnormalities of neurons involved in respiratory functions [78,79,80,81].

Similarly to what was described for cytokines, PGE2 can alter neuronal excitability and neurotransmission. This PG seems to be able to both enhance and inhibit glutaminergic transmission, modulate GABAergic receptor expression, and depress glynergic signaling pathways, although the exact mechanisms has not still clearly been identified; neuromodulation seems to be dependent on the type of PGE2 eicosanoid receptors (EPRs) enhanced [23,82,83,84,85,86].

In animal slices, PGE2 seems to be responsible for irregular breathing in vivo, and for inhibitory effects on respiratory centers in the brainstem modifying the respiratory rhythm and producing chemosensory responses [72,87,88]. Moreover, in fetal sheep, PGE2 seems to decrease respiratory frequency and induce hypoventilation, thus being responsible for the onset of apnea [89,90]. If directly injected at low concentrations (<200 nM) into the mouse pBOTC, PGE2 causes an increase of sigh frequency without any effect on eupneic breathing. High concentration of PGE2, however, seems to enhance eupneic breathing [91]. These opposing results on breathing functions may be context dependent.

Indomethacin injection in fetal sheep seems to cause COX inhibition by stimulation of breathing movements, showing that PGs are able to modulate respiratory activity [92].

PGE_2_ is implicated in various neuropathological conditions; this prostaglandin is able to bind to several G-protein coupled receptors: EP1R, EP2R, EP3R, and EP4R. This binding can result in distinct signaling pathways involved in neuronal injury, dysfunction, or protection, and may also improve or enhance inflammation as well as alter cerebral blood flow [5].

PGE_2_ interaction with EP1R increases the concentration of intracellular calcium with consequent excitotoxicity and neuronal death in mice [93,94]. Furthermore, EP1R activation stimulates vasoconstriction, reducing the cerebral flow and enhancing hypoxic-ischemic events [95].

The interaction between PGE_2_ and EP2R activates cAMP-response element binding (CREB). This is an important transcription factor that induces neuroprotection and synaptic plasticity [96,97,98,99]. In EP2R knockout mice, PGE_2_ binding to the EP2R has neuroprotective effects in cerebral ischemia and excitotoxicity, and middle cerebral artery occlusion [100,101]. Nevertheless, microglial EP2R deletion abrogates COX-2 and iNOS-mediated neurotoxicity after LPS injection [102]. It appears that the effects of the binding of PGE_2_ to EP2R may be context dependent, but in circumstances of systemic inflammation, the activation of the EP2R may have a deleterious role in the brainstem [5].

EP3R has shown the greatest affinity for PGE_2_ and has previously been involved in neuronal dysfunction and inflammation [87,103,104,105]. PGE_2_ binding to EP3R reduces the secretion of cyclic adenosine monophosphate (cAMP) and increases intracellular calcium, which is important for neuronal excitability and firing rate [106,107]. PGE_2_ production is mediated by IL-1b; in rats, PGE_2_ binding to EP3R reduces excitatory vagal neuronal signaling to the NTS [85]. This seems to impact peripheral chemosensory signaling to the brainstem.

Finally, EP4R is highly expressed not only in the brainstem, but also in the hypothalamus, and its functions are similar to that described for EP2R [108]. In rats and in EP4R knockout mice, PGE_2_ signaling after binding to EP4R may modulate anti-inflammatory and pro-inflammatory pathways [109,110]. EP4R is sited in the NTS and ventrolateral medulla of the brainstem, and systemic IL-1b injection seems to increase its expression [111]. However, further studies are required to determine its role in injuries caused by chorioamnionitis.

### 4.4. Effects of Chorioamnionitis on Peripheral Airways and Lungs

Animal studies clearly show that intrauterine inflammation is responsible for abnormalities in the development of fetal lungs. In rat’s fetal lung, intra-amniotic administration of IL-6 and IL-8 increases the expression of mRNA for surfactant proteins (SP) A, B, and C [112]. The increased production of surfactant proteins seems to be responsible for an increase in type 2 alveolar epithelial cells; in vivo and in vitro studies of fetal mice show that intra-amniotic LPS injection increases type 2 alveolar epithelial cell numbers [112].

In the fetal lungs of rabbits and sheep, intra-amniotic or intratracheal injection of IL-1b or LPS resulted in increased mRNA expression of SP-A and -B [113,114,115,116,117]. It was recently demonstrated that the fetal lung response to intrauterine inflammation is partly mediated by prostaglandins [117].

In sheep, intrauterine inflammation causes pulmonary inflammation with increased mRNA levels of IL-1b, IL-6, and IL-8, and chemokine inducible protein-10 (IP-10) within 24 h after the onset of inflammatory processes [118,119]. After 1–4 days LPS injection, reduced expression was observed for microvascular markers (vascular endothelial growth factor (VEGF), VEGF receptor 2, endothelial nitric oxide synthase (eNOS), tyrosine protein kinase receptor (Tie-2), and platelet endothelial cell adhesion molecule (PECAM) [119].

Alterations to fetal pulmonary vascular development induced by inflammatory processes are characterized by smooth muscle hypertrophy and deposition of collagen in the adventitial layer of pulmonary resistance arterioles [119]. Woldesenbet and Perlman observed that after LPS exposure by intra-amniotic injection, the above-mentioned alterations to the pulmonary vasculature, with an increase in pulmonary vascular resistance and subsequent reduction in pulmonary blood flow, occur in the fetus at days 2 and 4 [120].

In sheep, structural remodeling of the airspace occurs 7 days after LPS intra-amniotic exposure, resulting in 30% increase in alveolar volume, 20% decrease in alveolar number, and thinning of the alveolar epithelial surface [114].

Vascular remodeling of the fetal lungs after the onset of inflammatory processes is associated with persistent pulmonary hypertension of the newborn (PPHN). PPHN is characterized by increased resistance to pulmonary blood flow and right-to-left shunting through the foramen ovale (FO) and ductus arteriosus (DA), with consequent reduced left ventricular output [120,121,122]. Preterm lambs, when exposed to a single LPS injection via the amniotic fluid, 7 days prior to delivery, exhibit increased pulmonary vascular resistance and right-to-left shunting of blood across the DA within 30 min after delivery [123,124]. LPS exposure 2 or 4 days prior to delivery of preterm lambs does not have an important impact on pulmonary hemodynamics, suggesting that the full extent of vascular remodeling by that time has not still occurred [123,124]. Considering the remodeling of the pulmonary vascular and alveolar surface in fetal lambs exposed to intra-amniotic LPS [114], an etiologic link between chorioamnionitis, bronchopulmonary dysplasia, and PPHN may be suggested.

Table 2 summarizes the effects of pro-inflammatory cytokines and consequent inflammation on lungs and peripheral airways.

## 5. Conclusions

Preterm birth is associated with brainstem suboptimal development and modification of peripheral airways and lungs. These alterations are responsible for reduced respiratory control and apnea.

There is robust evidence confirming that chorioamnionitis is strongly associated with preterm birth and is responsible for an increased risk and severity of respiratory complications. There is extensive literature on brain injuries secondary to chorioamnionitis, which is also associated with vascular and pulmonary alterations. However, the effects of inflammation on brainstem respiratory nuclei still remain unclear.

Inflammation is associated with increased pro-inflammatory cytokines and prostaglandins synthesis, thus leading to functional changes of respiratory-related neurons within the brainstem. Dysregulation and damage to these respiratory neurons may be involved in the multifaceted pathophysiology of neonatal apnea in preterm neonates.

Understanding how chorioamnionitis may affect central respiratory nuclei and peripheral airways and lungs may be helpful in proposing effective therapeutic interventions within the delivery room, in order to reduce and prevent brain and lung injury, and to preserve neural circuitry responsible for the respiratory rhythm and coordinated functions.

Unfortunately, literature data focuses on animal models. Therefore, more clinical studies with clinical trials measuring inflammation in preterm babies born after chorioamnionitis are mandatory to better understand the role of inflammation on prematurity and neonatal apnea.

## Figures and Tables

**Table 1:** Resume of proinflammatory cytokines’ actions on the brainstem respiratory centers and modification of the respiratory activity. Abbreviations as used in the text.

Author (Reference No.)	Year of Publication	Population Studied	Cytokines Studied	Effect on the Brainstem
Balan et al. [54]; Ramirez et al. [55];	2012 2016	Animal models	IL-1b IL-6	Depresses inhibitory synaptic transmission Elevates excitatory signaling in the pBOTC Causes prolonged inspiratory drive and absent respiratory activity that leads to apnea
Galic et al. [57]; Vezzani et al. [56];	2012 2015	Animal models	IL-1b IL-6 TNF-a	Potentiates excitatory signaling in the CNS. Depresses inhibitory transmission in the CNS. Alters neuronal excitability through post-translational modification of GABAergic, glutaminergic, and glycinergic receptors, ultimately affecting synaptic plasticity and neurotransmission.
Wang et al. [58]; Wang et al. [59]; Galic et al. [57]; Vezzani et al. [56];	2000 2007 2012 2015	In vitro Animal models	IL-1b	Alters neuronal function in a concentration-dependent manner: *at low concentration* inhibits voltage-gated calcium currents, and lowers intracellular calcium concentrations (thus decreasing neurotransmitter release); *at high concentrations* increases inotropic NMDA receptors expression, and reduces transmission at GABAergic and glycinergic receptors in rat cerebellar and hippocampal cultures.
Beattie et al. [60]; Fourgeaud L et al. [61]; Galic et al. [57];	2002 2010 2012	Animal models	TNF-a	Alters neuronal excitability through the upregulation of AMPA and NMDA receptors, and induces GABA receptor endocytosis, as observed in rat hippocampus and cerebellum. This causes an increase in excitatory output (and possibly excitotoxicity), as well as a decrease in inhibitory signaling.
D’Arcangelo et al. [63]; Conroy et al. [62]; Wang et al. [58]; Vereyken et al. [64];	2000 2004 2007 2007	Animal models	IL-6	Reduces metabotropic glutamate receptor expression. Induces excitotoxicity by excessively activating NMDA receptors. Decreases GABAergic and glycinergic neurotransmission in dorsal horn neurons of rat spinal cord.
Gresham et al. [71]; Siljehav et al. [72].	2011 2014	Animal models	IL-1b	IL-1b injection into the rat NTS increases inspiratory time as early as 20 min post-application, with associated delayed respiratory activity (usually observed after 80 min). The systemic administration of IL-1b causes a decrease in respiratory frequency and induces apneustic episodes. Intrapulmonary and intraperitoneal injection of IL-1b to animal models attenuates hypoxic and hypercapnic responses, suggesting that chemosensory reflexes are impaired in response to inflammatory stimuli.

**Table 2:** Effects of proinflammatory cytokines and LPS-induced inflammation on lungs and airways. Abbreviations as used in the text.

Author (Reference No.)	Year of Publication	Population Studied	Cytokines Studied	Effect on Lungs and Airways
Ikegami et al. [112]	2000	Animal study	IL6 IL8	Intra-amniotic administration increases the expression of messenger RNA for surfactant proteins (SP) A, B, and C in the fetal lung; The increase in surfactant protein production has been suggested to be associated with an increase in type 2 alveolar epithelial cells.
Bry et al. [114] Willet et al. [115] Moss et al. [116] Kallpur et al. [117] Prince et al. [113]	1997 2000 2002 2003 2004	Animal studies	IL-8	Intra-amniotic or intratracheal injection of IL-1b or LPS resulted in increased mRNA expression of SP-A and -B in the fetal lungs.
Kallpur et al. [118] Galinsky et al. [119]	2004 2013	Animal studies	IL-1 IL-6 IL-8 Chemokin IP-10MIG	Intrauterine inflammation causes pulmonary inflammation with increased mRNA levels of inflammatory cytokines IL-1b, IL-6, IL-8, chemokines IP-10, and monokine induced by interferon gamma (MIG) within 24 h.
Bry et al. [114] Woldesenbet et al. [120] Polglase et al. [123] Polglase et al. [124] Galinsky et al. [119]	1997 2005 2010 2012 2013	Animal studies	LPS	Reduced expression of microvascular markers (vascular endothelial growth factor (VEGF), VEGF receptor 2, endothelial nitric oxide synthase (eNOS), tyrosine protein kinase receptor (Tie-2), and platelet endothelial cell adhesion molecule (PECAM)) occurs between 1 and 4 days after LPS exposure. These alterations to the pulmonary vasculature are associated with an increase in pulmonary vascular resistance and subsequent reduction in pulmonary blood flow in the fetus at 2 and 4 days, respectively, after intra-amniotic LPS exposure. Preterm lambs exposed to a single injection of intra-amniotic LPS 7 days prior to delivery showed increased pulmonary vascular resistance and right-to-left shunting of blood through the DA within 30 min after delivery. LPS exposure 2 or 4 days prior to delivery does not have such profound effects on pulmonary hemodynamics of preterm lambs, suggesting that the full extent of vascular remodeling had not occurred by that time. Considering the pulmonary vascular and alveolar remodeling demonstrated in fetal lambs exposed to intra-amniotic LPS [114], a causative link between chorioamnionitis, BPD, and PPHN becomes increasingly apparent.

## Data Availability

Data are available on request to the corresponding author.
